# Changes in visual function and retinal structure in the progression of Alzheimer's disease

**DOI:** 10.1371/journal.pone.0220535

**Published:** 2019-08-15

**Authors:** Elena Salobrar-García, Rosa de Hoz, Ana I. Ramírez, Inés López-Cuenca, Pilar Rojas, Ravi Vazirani, Carla Amarante, Raquel Yubero, Pedro Gil, María D. Pinazo-Durán, Juan J. Salazar, José M. Ramírez

**Affiliations:** 1 Instituto de Investigaciones Oftalmológicas Ramón Castroviejo, Facultad de Medicina, Universidad Complutense de Madrid, Madrid, Spain; 2 Departamento de Inmunología, Oftalmología y ORL, Facultad de Medicina, Universidad Complutense de Madrid, Madrid, Spain; 3 Departamento de Inmunología, Oftalmología y ORL, Facultad de Óptica y Optometría, Universidad Complutense de Madrid, Madrid, Spain; 4 Servicio de Oftalmología, Hospital Universitario Gregorio Marañón, Madrid, Spain; 5 Unidad de Memoria, Servicio de Geriatría, Hospital Clínico San Carlos, Madrid, Spain; 6 Unidad de Investigación Oftalmológica «Santiago Grisolia»/FISABIO, Valencia, Spain; 7 Grupo de Oftalmobiología Celular y Molecular, Facultad de Medicina y Odontología, Universidad de Valencia, Valencia, Spain; Doheny Eye Institute/UCLA, UNITED STATES

## Abstract

**Background:**

Alzheimer’s Disease (AD) can cause degeneration in the retina and optic nerve either directly, as a result of amyloid beta deposits, or secondarily, as a result of the degradation of the visual cortex. These effects raise the possibility that tracking ophthalmologic changes in the retina can be used to assess neurodegeneration in AD. This study aimed to detect retinal changes and associated functional changes in three groups of patients consisting of AD patients with mild disease, AD patients with moderate disease and healthy controls by using non-invasive psychophysical ophthalmological tests and optical coherence tomography (OCT).

**Methods:**

We included 39 patients with mild AD, 21 patients with moderate AD and 40 age-matched healthy controls. Both patients and controls were ophthalmologically healthy. Visual acuity, contrast sensitivity, colour perception, visual integration, and choroidal thicknesses were measured. In addition, OCT and OCT angiography (OCTA) were applied.

**Findings:**

Visual acuity, contrast sensitivity, colour perception, and visual integration were significantly lower in AD patients than in healthy controls. Compared to healthy controls, macular thinning in the central region was significant in the mild AD patients, while macular thickening in the central region was found in the moderate AD group. The analysis of macular layers revealed significant thinning of the retinal nerve fibre layer, the ganglion cell layer and the outer plexiform layer in AD patients relative to controls. Conversely, significant thickening was observed in the outer nuclear layer of the patients. However, mild AD was associated with significant thinning of the subfovea and the nasal and inferior sectors of the choroid. Significant superonasal and inferotemporal peripapillary thinning was observed in patients with moderate disease.

**Conclusions:**

The first changes in the mild AD patients appear in the psychophysical tests and in the central macula with a decrease in the central retinal thickness. When there was a disease progression to moderate AD, psychophysical tests remained stable with respect to the decrease in mild AD, but significant thinning in the peripapillary retina and thickening in the central retina appeared. The presence of AD is best indicated based on contrast sensitivity.

## Introduction

Alzheimer's disease (AD), a neurodegenerative disorder related to age, is produced by a neuronal loss and synapses in the cerebral cortex [1]. The extracellular plaque forming deposits of the protein amyloid-β (Aβ) and the intraneuronal accumulations of the hyperphosphorylated microtubule-associated protein tau, which forms the neurofibrillary tangles [2], characterize this disease. In addition to age, other risk factors include genetic alterations, such as mutations in the gene that encodes the precursor of Aβ protein or the presence of APOE ε4 allele [3,4].

AD is the most common type of dementia in the world [5] contributing to 60–70% of all cases [6]. In the last few decades, there have been important advances in the diagnosis of AD. The retina is a projection of the central nervous system (CNS), and its peripheral location can provide an accessible and non-invasive path leading to the examination of brain pathology [7,8]. Due to these characteristics, the visual and structural tests used in the diagnosis of ophthalmological diseases can be used as valuable tools to confirm the diagnosis of neurodegenerative diseases.

Due to the irreversible course and the absence of an effective treatment for AD, it is essential to perform the diagnosis at an early stage. In addition, patients should be properly classified according to each stage of their disease. Within the test that value these capabilities, Folstein, in 1975, developed the Mini Mental State Examination (MMSE) Folstein et al 1975 [9] which meant an enormous contribution to the assessment of AD patients compared to other short test that explored only short-term memory. The MMSE explores five areas: orientation, registration, attention and calculation, and memory and language. Based on these two premises, namely early diagnosis and MMSE, we have classified our AD patients into two groups: mild AD (25.18±3.80) and moderate AD (19.89±2.76). The study aimed to:

- analyse the changes that take place in the mild AD group, and the moderate AD group by using psychophysical and structural tests,
- compare these two groups with a population of healthy age-matched controls,
- assess whether there was a progression in these AD groups.

To the best of our knowledge, few studies analyse a plethora of tests and their prognostic value in the two early stages of AD.

## Methods

### Subjects

**Fig 1:**
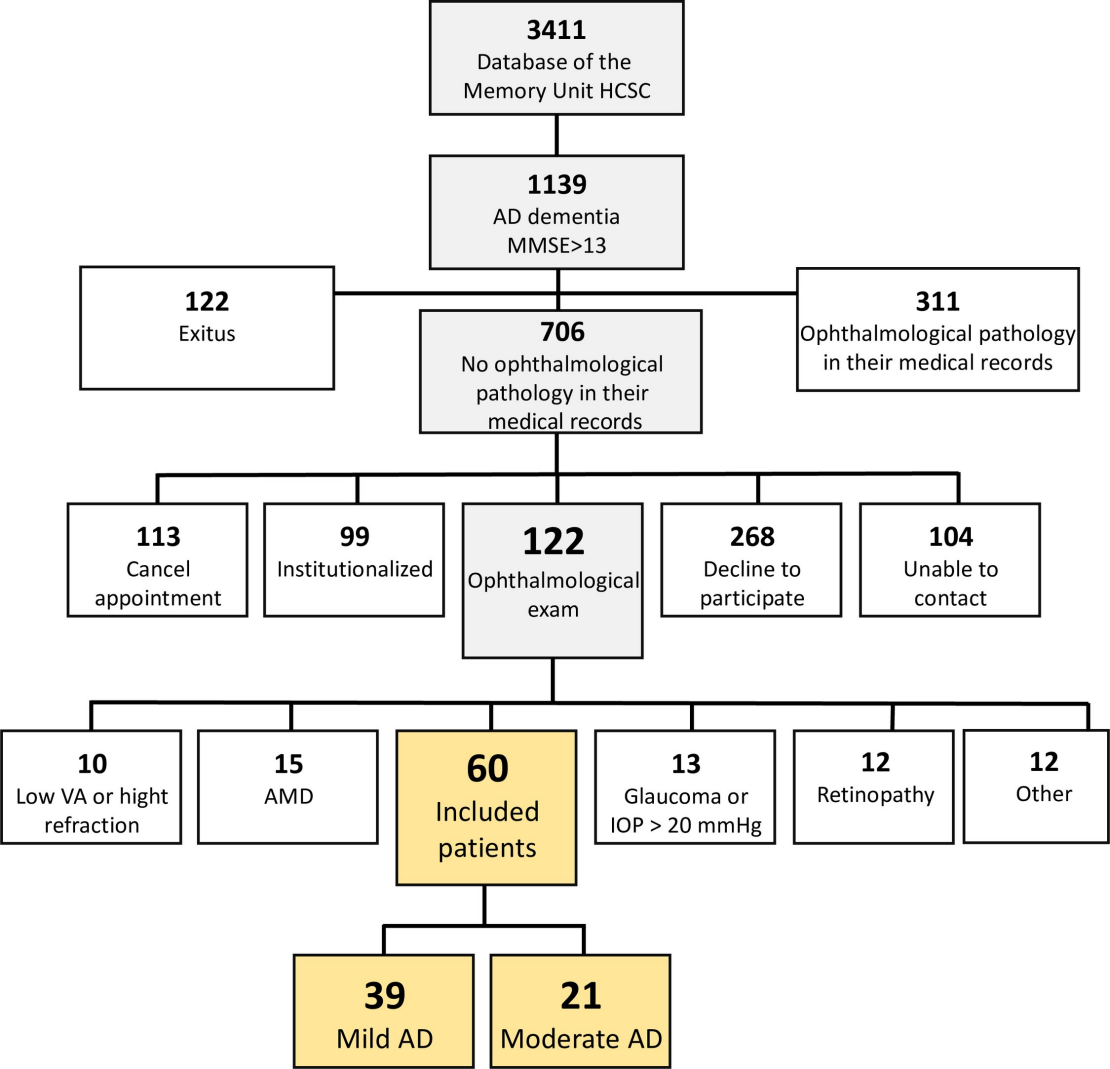
Flow diagram patient's inclusion.

To select patients, we reviewed the Database of the Memory Unit of the Hospital Clinico San Carlos in Madrid (Spain), consisting of a total of 3411 patients (Fig 1). We first excluded the patients with an MMSE lower than 17 followed by those with a mood or psychiatric disorder. Next, we considered patients with mild AD and moderate AD. These patients, according to the National Institute of Neurological and Communicative Disorders and Stroke-AD and Related Disorders Association and the Diagnostic (NINCDS-ADRDA) and Statistical Manual of Mental Disorders IV (DSM IV), had mild and moderate cognitive impairments according to the Clinical Dementia Rating scale (CDR). Then, ophthalmic medical records of these patients, excluding those who were previously diagnosed with an ophthalmological pathology (glaucoma or suspected glaucoma, media opacity, and retinal diseases) were reviewed. After this analysis, 122 patients with AD satisfied all the requirements (having an MMSE of over 17 and being free of ocular disease and systemic disorders affecting vision in their medical record) needed to participate in the study. Aged-matched control subjects were healthy controls from the Geriatric Unit of the Hospital Clínico San Carlos (Madrid) (n = 37) or the relatives of AD patients (husband or wife) with no history of memory decline and MMSE>25 (n = 3). Of the 122 AD patients and 60 age-matched control subjects selected, 62 AD patients and 40 age-matched control subjects were subsequently excluded due to posterior pole pathology, which included macular degeneration, drusen, suspicion of glaucoma, glaucoma, epiretinal membrane, or cataracts, all of which prevented ocular examination. Because of this selection, 39 patients with mild AD, 21 patients with moderate AD and 40 age-matched control subjects were considered for the study. All patients had adequate reasoning faculties and be in possession of all relevant facts to give the written informed consent. Written informed consent was obtained from these three groups. The research followed the tenets of the Declaration of Helsinki, and the protocol was approved by the local ethics committee (CEIC Hospital Clinico San Carlos 11/372-E). In S1 File there are all the study's underlying data set.

### Methods

The clinical evaluation of our AD included a review of the medical records, a caregiver interview, physical and neurological examinations, a psychometric test, neuroimaging techniques, and routine laboratory testing for dementia. In the ophthalmological part of the study, only one eye of each patient was analysed. All participants met the following inclusion criteria: being free of ocular disease, having AREDS Clinical Lens Standards affecting vision, having a best-corrected visual acuity (VA) of 0.5 dec, having less than ±5 spherocylindrical refractive error, and having intraocular pressure of less than 20 mmHg. For screening, all AD patients and control subjects underwent a complete ophthalmologic examination, including VA, refraction, anterior segment biomicroscopy, applanation tonometry (Perkins MKII tonometer), CS test CSV-1000E, Roth 28-hue colour test, a perception digital test (PDT) [10], a dilated fundus examination and an OCT.

### Visual acuity

The monocular best-corrected VA was determined using a standard clinical Snellen eye chart (decimal scale). The patients started to read each row from the top of the Snellen eye chart and proceeded towards the bottom. This process ended when the hit rate was less than five of eight (an approximation of 56.25%, the steepest point of the psychometric acuity function).

### Contrast sensitivity function

The CS test was performed with the CSV-1000E system (VectorVision, Greenville, OH, USA) and in the presence of the best-corrected VA for far vision. The patient was encouraged to guess whether a grating was at least partially visible as the threshold was approached. The contrast level of the last correct response was recorded as the threshold.

### Colour perception

To administer a colour-vision test that did not require a naming response, we used Roth 28-Hue Color Test (Luneau, Paris), a quick and easy colour arrangement test first described by Roth [11]. Colour perception was assessed in the presence of best-corrected VA for near vision. Test instructions were repeated by the examiner during the test when necessary. The time to perform the test was not restricted, and the subject was allowed to make corrections. Errors classified the observer as protanomalous, deuteranomalous, or tritanomalous (red-, green-, or blue-deficient, resp.). The manufacturer’s manual blue axis errors were considered when caps 43 to 64 were malpositioned and deutan axis errors were between caps 42 to 85. Thus, the tritan and deutan errors were quantified.

### Perception Digital Test (PDT)

The PDT [10], is an easy, fast, and sensitive method for evaluating disorders of visual perception in mild AD patients. The test includes 15 sheets. Each sheet shows the same picture at different positions in space. The pictures are distorted by choice of special effects: geometric effects (tile) and the effect of the frame 24/48 of the MGI Photo Suite III program. The patient had to identify the picture that was properly oriented in space.

### Optical coherence tomography (OCT)

The macular thickness retinal and peripapillary nerve fiber layer (RNFL) thickness were measured by the OCT Model 3D OCT-1000 (Topcon, Japan) and the thickness of the macula was segmented into layers by OCT Spectralis (Heidelberg Engineering, Heidelberg, Germany), after pupil dilatation in both cases. The RNFL thickness was scanned 3 consecutive times per patient in each area studied. The mean values were considered for statistical analysis. All tests were performed by the same optometrist (ESG) under the same conditions.

**Fig 2:**
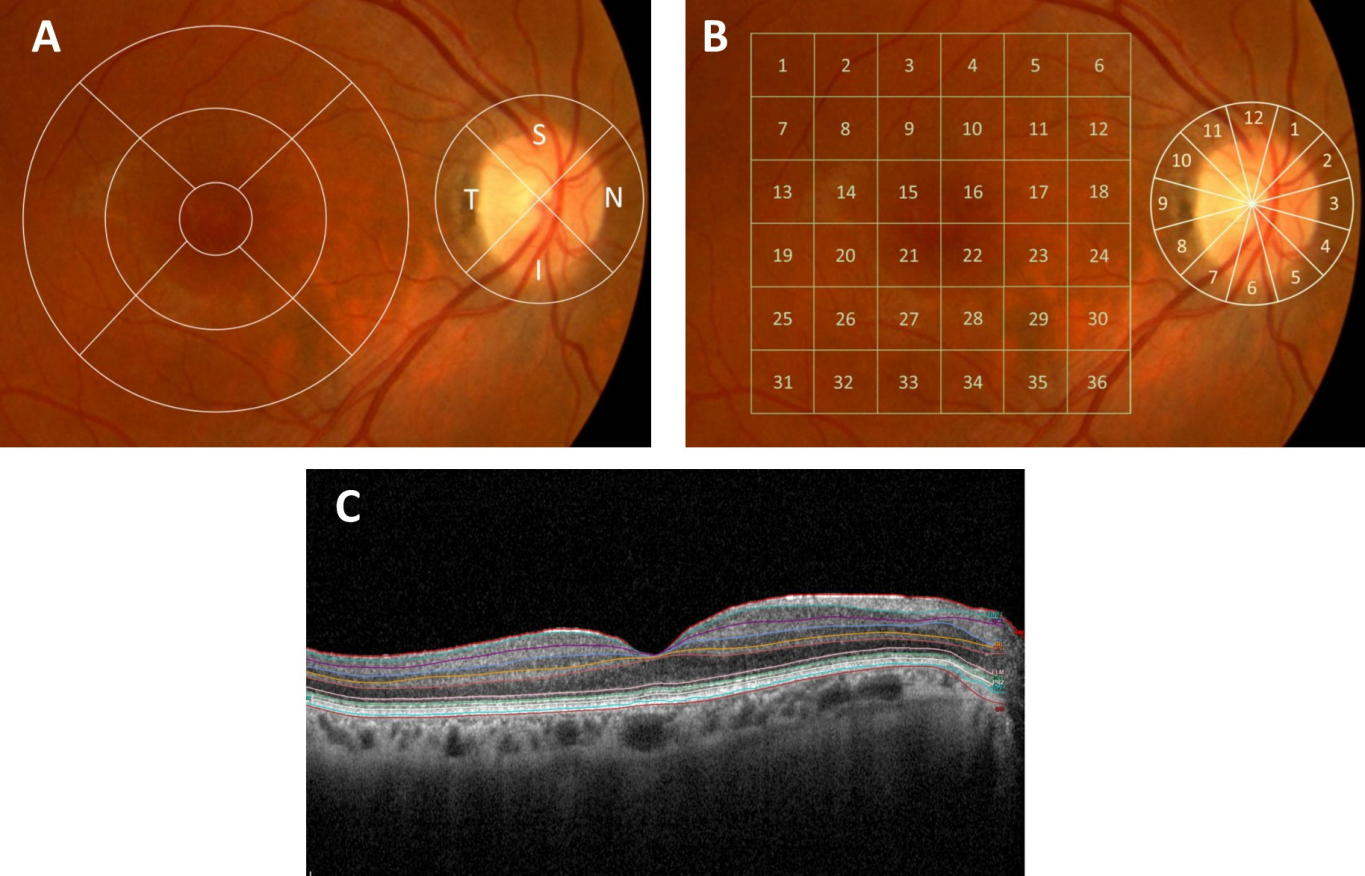
OCT report of retinal thickness analysis. (A) Concentric macular rings and four peripapillary quadrants (B) Rectangular grid 6x6 macular sectors and peripapillary thickness for each 12-o’clock hour and (C) Macular thickness segmentation of 10 retinal layers.

The macular thickness was evaluated in two ways: by concentric circular rings and by a rectangular grid with 6x6 sectors. First, the macular RNFL thickness data were displayed in three concentric rings centred in the foveola. They were distributed as they appear in Fig 2A. The total volume of the macula as provided by the OCT was also calculated. The analysis of the macula using a 6x6 mm grid was performed by centring a grid in the fovea with a scanning density of 512 x 128 pixels in 3.5s (27,000A scan/s). In total, 36 squares were analysed (1 mm by 1 mm each). The centre of the macula was defined as the thinnest point of the foveal depression. The average of each square was noted for analysis (Fig 2B).

The peripapillary RNFL thickness parameters evaluated in this study were: i) average thickness (360° measurement); ii) thickness for the four peripapillary quadrants (μm) (Fig 2A) and iii) thickness for each 12-o’clock hour position with the 3-o’clock position as nasal, 6-o’clock position as inferior, 9-o’clock position as temporal, and 12-o’clock position as superior (Fig 2B).

The Spectralis OCT software enables the automatic segmentation of the layers of the retina and the quantification of the thickness of each layer through the same segmentation of concentric circular sectors mentioned above. After collecting the images, a manual check was performed to verify that the fovea was well placed and that the automatic division of the layers was done correctly. If this procedure failed, the same experienced examiner performed the layer segmentation manually (Fig 2C).

The good scan criteria were determined as the signal-to-noise ratio >30 and accepted A-scans >95% in fast RNFL scanning. All measurements were given in microns, according to the calibration provided by the manufacturers and the total volume was recorded in mm^3^.

### Statistical analysis

Data for the statistical analysis were introduced and processed in SPSS 19.0 (SPSS Inc., Inc., Chicago, IL, USA). The data were reported as median values ± interquartile range. The differences between different study groups (Mild AD, moderate AD and control eyes) were analysed using the Mann-Whitney test. Sensitivity was set at 90% specificity, and the area under the receiver operator characteristic (aROC) analysis was used to discriminate between the healthy and the mild AD patients. These data were calculated for all the psychophysical tests analysed and for the OCT analysis. The association between the tests and the MMSE was evaluated by the Pearson’s correlation coefficient. A *P* value of ≤ 0,05 was considered statistically significant.

## Results

In the mean MMSE values, there were significant differences between the 3 study groups (28,58 ± 1,83, control; 25,18 ± 3,80, mild AD; 19,89 ± 2,76, moderate AD; p <0.001, in all cases). The rest of the data analysed as IOP, gender and race did not present significant differences between the study groups.

### Psychophysical tests

#### Visual acuity

**Fig 3:**
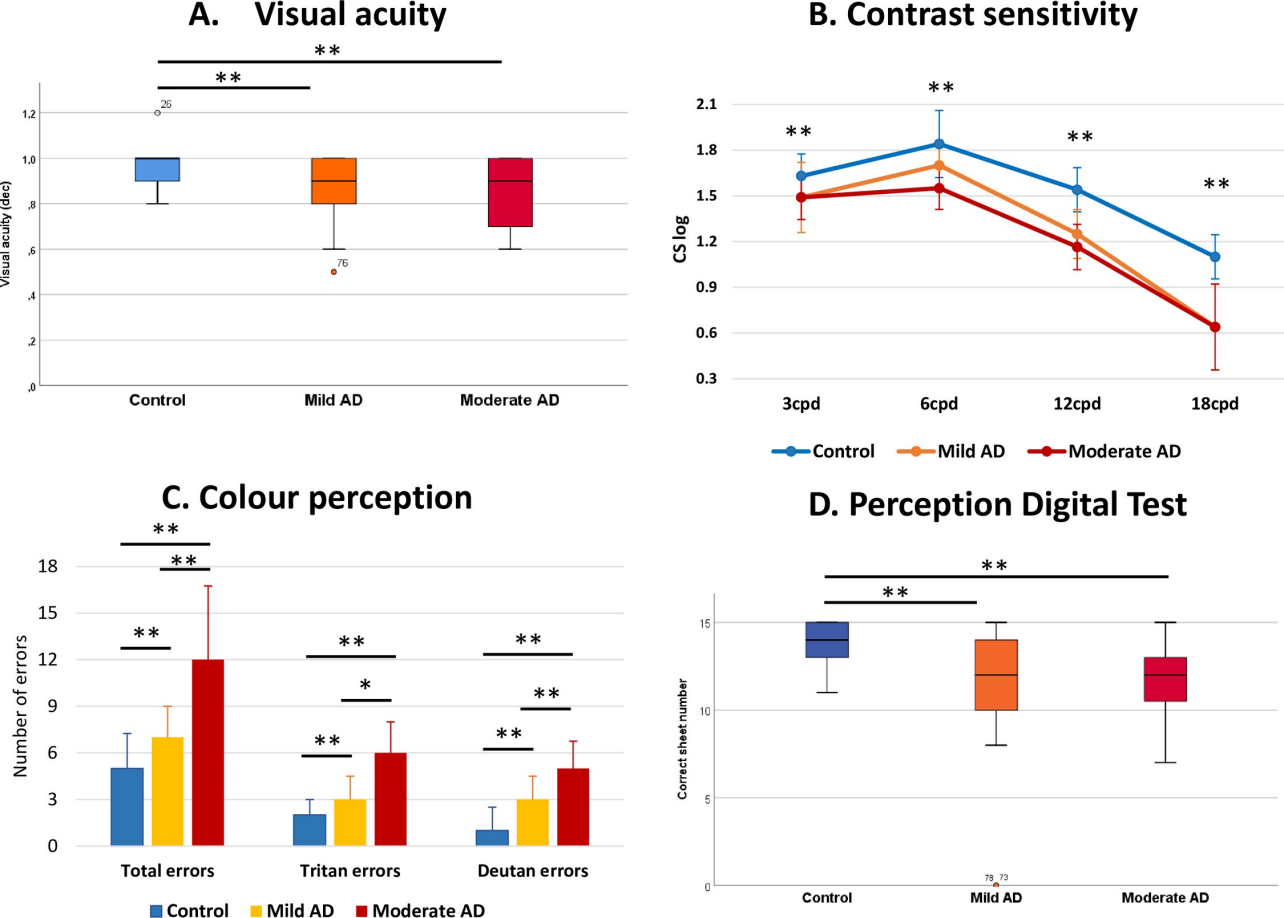
Median data of the psychophysical tests. (A) Visual acuity, (B) contrast sensitivity, (C) desaturated Rue 28-hue color test, and (D) perception digital test. Each bar represents the median ± interquartile range. * *P* < 0.05 versus control. ** *P* < 0.01 versus control. Mann-Whitney *U* test.

The median VA both in the mild AD patients and moderate AD patients significantly decreased in comparison with the age-matched control group (p<0,001) (Table 1, Fig 3A).

**Table 1:** Median data and p-value of the psychophysical tests.

		Control	Mild AD	Moderate AD	Mild AD vs control		Moderate AD vs control		Moderate AD vs Mild AD	
		(n = 40)	(n = 39)	(n = 18)	% difference	P-value	% difference	P-value	% difference	P-value
**Visual acuity (dec)**	** **	1.00 ± 0.10	0.90 ± 0.20	0.90 ± 0.30	-10.00.	<0.001**	-10,00	0.003**	0.00.	0.921
**Constrast sensitivity (cpd)**	**3**	1.63 ± 0.29	1.49 ± 0.46	1.49 ± 0.29	-8.59.	<0.001**	-8,59	0.009**	0.00.	0.422
	**6**	1.84 ± 0.44	1.70 ± 0.33	1.55 ± 0.28	-7.61.	<0.001**	-15,76	<0.001**	-8.82.	0.373
	**12**	1.54 ± 0.29	1.25 ± 0.32	1.16 ± 0.30	-18.83.	<0.001**	-24,68	<0.001**	-7.20.	0.599
	**18**	1.10 ± 0.29	0.64 ± 0.56	0.64 ± 057	-41.82.	<0.001**	-41,82	<0.001**	0.00.	0.781
**Number of errors**					** **	** **	** **	** **	** **	** **
** **	**Total**	5.0 ± 5.0	7.0 ± 4.0	12.00 ± 8.0	40.00.	0.034*	140.00.	<0.001**	71.43.	0.001**
	**Tritan**	2.0 ± 2.0	3.0 ± 3.0	6.0 ± 5.0	50.00.	0.002**	200.00.	<0.001**	100.00.	0.017*
	**Deutan**	1.0 ± 3.0	3.0 ± 3.0	5.0 ± 3.0	200.00.	0.003**	400.00.	<0.001**	66.67.	0.002**
**PDT**		14.00 ± 2.00	12.00 ± 4.00	11.50 ± 3.0	-14.29.	<0.001**	-17.86.	<0.001**	-4.17.	0.650

Median ± interquartile range

*P<0.05

**P<0.01 Mann- Whitney U test

[AD, Alzheimer’s disease; vs: versus; dec: decimal scale; cpd: cicles per degree; PDT: perception digital test]

#### Contrast sensitivity

The analysis of CS of the mild AD and moderate AD patients revealed a statistically significant reduction at all spatial frequencies tested (3, 6, 12, and 18 cpds) in both groups in comparison with age-matched control subjects (p<0,001; in all instances). In addition, the results of both groups showed that the spatial frequency was higher when the loss of CS perception was the greater. Thus, in comparison with the age-matched control group, the spatial frequency of 18 cpds showed the greatest CS decrease in the mild AD patients and the moderate AD patients (40.67% and 36.98% respectively) (Table 1, Fig 3B).

#### Colour perception

The analysis of the non-specific total errors in the Farnsworth test regarding the three groups of the study showed that there was a greater number of non-specific errors in both the mild AD patients and the moderate AD patients compared with the control group. In both groups, consisting of the mild and moderate patients, this difference was statistically significant (p<0,05; p<0,001 respectively). The increase in total errors amounted to 30.96% in the mild AD group and over 132.26%, the control group (Table 1, Fig 3C). When comparing the two groups with AD, more significant errors were found in patients with moderate AD than in patients with mild AD (p <0.001). The increase in errors in patients with moderate AD was 77.34% with respect to the age-matched controls (Table 1, Fig 3C).

The analysis of the tritan axis revealed that the number of tritan unspecific errors significantly increased in both the mild AD patients and the moderate AD patients in comparison with the age-matched control (p<0,001 in both cases). In addition, significantly more errors in the tritan axis were found in patients with moderate AD than in patients with mild AD (p <0.001). The increase in errors found in moderate AD patients corresponded to 49.04% compared to the age-matched controls (Table 1, Fig 3C).

The analysis of non-specific errors in the deutan axis showed that there were significant increases in the number of errors in both the mild AD patients and the moderate AD patients with respect to controls (p<0.05; p<0,001 respectively). This error increase corresponded to 59.89% in the mild AD patients and 158.49% in the moderate AD patients compared to the control. Finally, when comparing the two groups with AD, significantly more errors were found in the moderate AD group than in the mild AD group. The error increase in the moderate AD group amounted to 61.71% (Table 1, Fig 3C).

#### Perception Digital Test (PDT)

The PDT mean value found in the age-matched control group was significantly higher than the value of the mild AD group and the value of the moderate AD group (p <0.001 in both cases) (Table 1, Fig 3D).

### Optical coherence tomography

#### Macular thickness analysis by OCT

**Fig 4:**
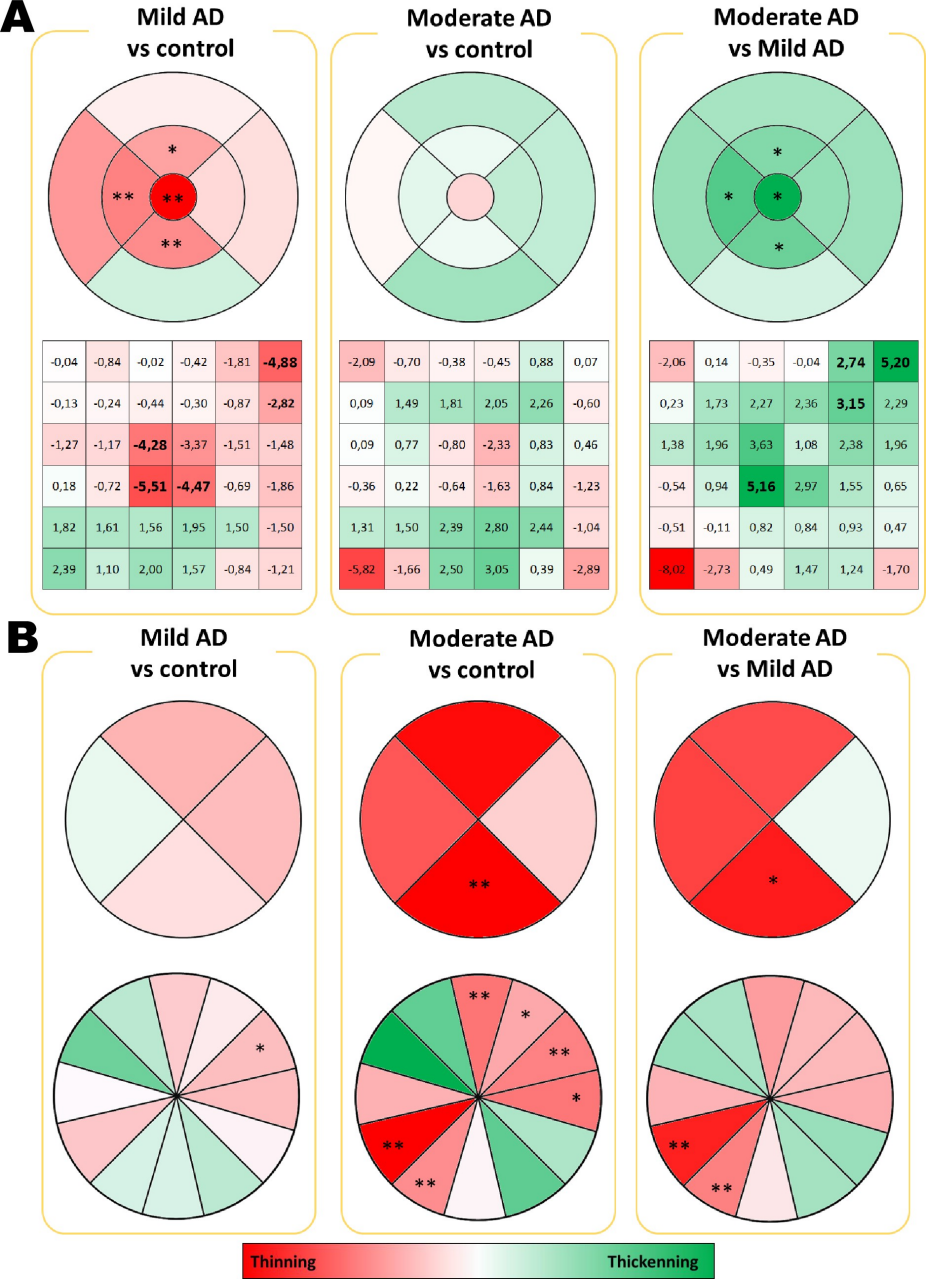
Colorimetric percentage differences of retinal thickness between groups. (A) Macular OCT. Upper: OCT concentric circular rings. Bottom: 6x6 mm rectangular grid, (B) Peripapillary OCT. Upper: Four peripapillary quadrants. Bottom: 12-o’clock hour position segmentation. In red, thickness decrease; in green thickening. In bold: *P < 0.05. **P < 0.01. Mann-Whitney *U* test.

In the patients with mild AD, both the thickness analysis of the circular sectors and the concentric sectors of the macular region revealed significant decreases. There were also significant decreases in the fovea thickness (p<0.01) and the sectors of the inner macular ring, superior (p<0.05), the inferior and temporal (p<0.01 in both cases) with respect to the age-matched controls (Fig 4A). However, in the eyes of the moderate the AD patients compared to the mild AD patients, there were significant increases in both the fovea thickness and the inner macular ring thickness in the superior, nasal and inferior sectors (p<0.05). The maximum increase was recorded in the fovea, being 7.1% thicker than those of the moderate AD patients (Fig 4A).

The macular quadrangular grid analysis in the eyes of the mild AD patients in comparison to age-matched controls showed a significant decrease in retinal thickness in squares 6 (p <0.05), 12 (p <0.05), 15 (p <0.01), 21 (p <0.01) and 22 (p <0.01) (Fig 4A). The greatest decrease in thickness corresponded to square 21 with a -5.5% decrease with respect to the age-matched control. The moderate AD patients with respect to the mild AD patients, showed a significant increase in retinal thickness in squares 5 (p <0.05), 6 (p <0.01), 11 (p <0.05), and 21 (p <0.05). The greatest increase in thickness corresponded to squares 6 and 21 with an increase of 5.2% regarding the mild AD patients (Fig 4A).

#### Peripapillary RNFL segmentation thickness analysis by OCT

When comparing the moderate AD patients with the age-matched controls, there was a significant decrease in the inferior peripapillary sector (p <0.01) and in the average thickness the moderate AD patients with respect to those of the control patients (p <0.05). When the moderate AD patients were compared with the mild AD patients, it was found that only the significant decrease in the lower peripapillary sector persisted (p <0.05). These results, with respect to the control, represented a decrease of 13.4% in the lower peripapillary sector in the eyes of the mild AD patients and a thickness decrease of 15% in the eyes of the moderate AD patients (Fig 4B).

When analysing the peripapillary retinal thickness in the 12 hourly sectors, we observed that only sector 2 in the eyes of the mild AD patients in comparison with those of the age-matched controls, showed a significant decrease, representing a 10.9% decrease in thickness. However, when analysing the moderate AD group with respect to the age-matched control group, it was observed that there was a significant thinning in sectors 1 (p <0.05), sector 2 (p <0.01), sector 3 (p <0.01), sector 7 (p <0.01), sector 8 (p <0.01) and sector 12 (p <0.01). Most of the thinning was shown in sector 8, the decrease in thickness of which represented -44.4% with respect to the age control group. The comparison of both the mild and moderate groups with AD showed that sectors 7 and 8 were significantly thinned in the moderate AD patients with respect to the mild AD patients. The results showed decreases in thickness of 21.7% and 38.6% respectively. (Fig 4B).

### Predictive value of the diagnostic methods. aROC curves

The aROC curve for psychophysical tests in AD patients showed that each test has a high predictive value (p<0.01). The test with the greatest prognostic value in AD patients was CS for 18 cpd and 12 cpd spatial frequencies, followed by CS for 6 cpd; PDT; unspecific errors in the deutan region; the tritan region; VA, CS for 3 cpd; and finally unspecific totals errors in Farnsworth test (Table 2).

**Table 2:** aROC analysis of psychophysical tests and OCT analysis in Alzheimer’s disease.

			aROC	SD	P-value
**Psychophysical tests**	**VA**		0,714	0,051	<0,001**
	**CS**	**3**	0,712	0,052	<0,001**
		**6**	0,795	0,046	<0,001**
		**12**	0,828	0,043	<0,001**
	** **	**18**	0,833	0,040	<0,001**
	**Color test**	**Total errors**	0,702	0,054	0,001**
		**Tritan errors**	0,736	0,051	<0,001**
		**Deutan errors**	0,746	0,051	<0,001**
	**PDT**		0,792	0,045	<0,001**
**Macular OCT**	**Fovea**		0,708	0,064	0,004**
	**Inner macular ring**	**Superior**	0,638	0,070	0,056
		**Nasal**	0,616	0,072	0,108
		**Inferior**	0,680	0,067	0,013*
		**Temporal**	0,665	0,068	0,022*
	**Rectangular grid 6x6 macular sectors**	**15**	0,681	0,066	0,012*
		**16**	0,663	0,067	0,024*
		**21**	0,726	0,063	0,002**
		**22**	0,707	0,065	0,004**

*P-value < 0.05

** P-value < 0.01

(aROC: area under the receiver operating characteristic; AD: Alzheimer’s disease; PDT: perception digital test; SD: standard deviation).

The aROC curve in the analysis of the macula by circular concentric sectors illustrated that the measures that showed statistical significance were the foveal thickness, followed by the lower sector and temporal of the inner macular ring. The rest of the parameters analysed did not show statistical significance (Table 2).

The aROC curve values that showed a significant predictive value in the analysis of the macular thickness by a quadrangular grid of 6x6 were the square 15, the square 16, square 21 and square 22 (Table 2).

In the aROC curves values for the 12 peripapillary hourly sectors were observed a statistical significance for sector 2 and sector 8 (Table 2).

### Analysis of Pearson's correlation with the values of MMSE

In the analysis of the Pearson's correlation between the MMSE scores and the psychophysical tests, it was found that all the tests had a statistical significance (p <0.001). The highest correlation with the MMSE was found in the CS test in the frequency of 6 cpd with a positive correlation of 0.544 (p <0.001), followed by nonspecific errors of the deutan axis in the analysis of colour perception with a significant inverse correlation of -0.506 (p <0.001) (a lower MMSE, more errors); the total errors (r = -0.483; p <0.001); the non-specific errors of the tritan axis (r = -0.451; p <0.01) that present very high correlations, followed by the SC for 12 cpg (r = 0.439; p < 0.01) and 18 cpg (r = 0.411; p <0.01), VA (r = 0.406, p <0.01), the SC for 3 cpg (r = 0.386, p <0.01) and TDP (r = 0.257); p <0.01), where in the latter of all positive correlations.

In the analysis of the correlations between the MMSE scores and the parameters of the OCT, significant correlations were found in both the measurements of the macular region and those of the peripapillary region. The highest correlation with the MMSE score and macular analysis through a 6x6 square grid, was found in square 4 (r = 0.277, p <0.05), followed by square 6 (r = 0.262, p <0.05), square 12 (r = 0.261, p <0.05), square 24 (r = 0.253, p <0.05), square 16 (r = 0.243, p <0.05), square 5 (r = 0.242, p <0.05), square 3 (r = 0.238, p <0.05), square 30 (r = 0.232, p <0.05) and in the concentric sectors analysis the temporal exterior macular ring (r = 0.229, p <0.05).

The highest to lowest significant correlations of the papillary analysis with the MMSE score were: in the analysis of hourly sectors, sector 12 (r = 0.370, p <0.01); in the analysis of the 4 sectors, in the superior sector (r = 0.340, p <0.01) and the inferior (r = 0.288, p <0.01), the average peripapillary thickness (r = 0.308, p <0.01) and finally in the analysis of 12 sectors the sector 7 (r = 0.278, p <0.01) and 1 (r = 0.260, p <0.01).

### The study of all the retinal layers thickness in the macular region through Spectralis OCT

#### Thickness of the retinal nerve fiber layer (RNFL) in the macular retina

**Fig 5:**
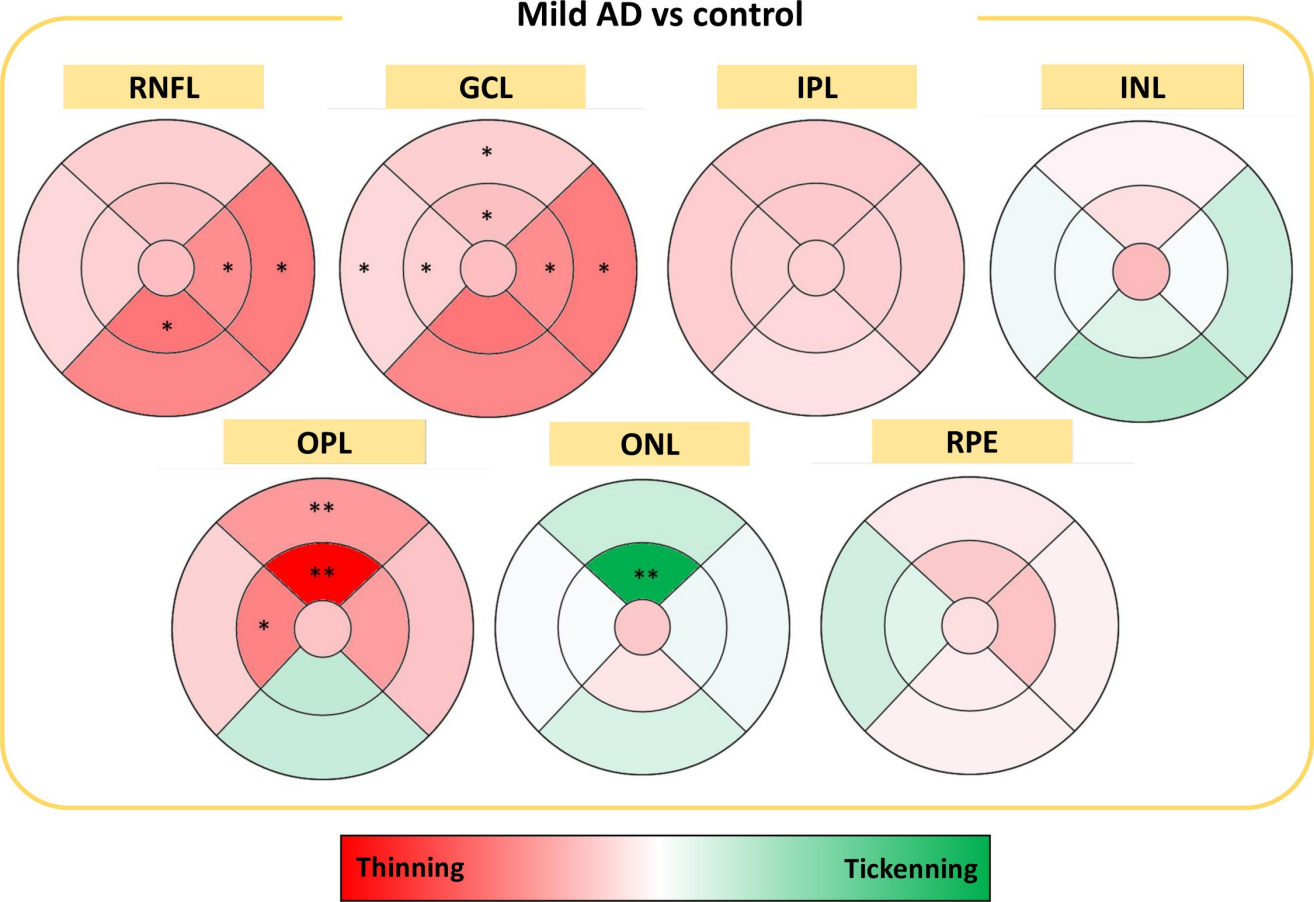
Colorimetric percentage differences of the all retinal layers between mild AD and control group. In red, thickness decrease; in green thickening. *P < 0.05. **P < 0.01. Mann-Whitney *U* test.

The eyes of the mild AD patients, compared to controls, showed a significant thinning of the RNFL in the inner macular ring in the nasal and inferior sectors, (p <0.05 in both cases) and in the outer macular ring in the nasal sector (p <0.05). The greatest decrease of thickness in the RNFL was in the inner macular ring in the lower sector (17.09%) (Fig 5).

#### Thickness of the ganglion cell layer (GCL) in the macular retina

In the GCL, the eyes of the mild AD patients with respect to those of the matched age controls showed a significant thickness decrease in the inner macular ring and in the outer macular ring, in the superior, nasal and temporal sectors (p <0.05 in all cases). In addition, a significant reduction in the total volume of the GCL was also found (p <0.05) (Fig 5).

#### Thickness of the inner plexiform layer (IPL) in the macular retina

In the IPL, there was a non-significant generalized thinning of this layer at the macular level (Fig 5).

#### Thickness of the inner nuclear layer (INL) in the macular retina

In the analysis of the INL, a slight decrease in thickness in the fovea and the superior sectors was observed. In the rest of the sectors, a slight increase in thickness predominated (Fig 5)

#### Thickness of the outer plexiform layer (OPL) in the macular retina

The OPL of the mild AD patients compared to the matched-age controls showed a significant thickness decrease in the inner macular ring in the superior sector and the temporal sector (p <0.01, p <0.05 respectively), in the outer macular ring in the upper sector, and in the total volume of the OPL (p <0.01, p <0.05 respectively) (Fig 5)

#### Thickness of the outer nuclear layer (ONL) in the macular retina

In the ONL, the mild AD patients showed a significant increase in the inner macular ring in the superior sector (p <0.01) with respect to the matched-age controls (Fig 5).

#### Thickness of the retinal pigment epithelium (RPE) layer of the macula

In the analysis of the EPR layer, no significant differences were found in any of the sectors analysed with respect to the age-matched control (p> 0.05) (Fig 5).

## Discussion

To the best of our knowledge, few studies analyse a plenitude of tests and their prognostic values in the two early stages of AD. One of the noteworthy points of the present study was the careful selection of the samples. None of the three study groups showed abnormalities or eye diseases that could mask the results. The sample of patients with AD was very homogeneous in terms of age, stage of disease, ethnicity, and educational attainment. Patients with mild AD had high MMSE scores (25.18 ±3.80), higher than any value reported in the different studies [12–16]. These patients were in a very early stage of the disease. Patients with moderate AD had MMSE scores (19.89 ± 2.76), which allowed them to understand the instructions to perform the tests correctly. The tests were chosen by taking into account the cognitive decline and possible nominative deficit. VA tests were performed by isolating the letters since they were dissipated that way, patients with dementia had better values [17]. To measure the CS, we used the CSV-1000E, with which the patients do not need to verbalize the result. Therefore, the results were more independent of the VA values [13]. The analysis of the colour perception was carried out with the Roth 28-hue test since verbalization was not necessary for its realization. In the measurements of the retinal thickness by OCT, the patient collaboration was minimal, and the patient was only required to keep an eye open and look fixedly at the fixation stimulus.

The VA in our patients with mild AD and moderate AD showed a significant decrease with respect to the control group. In addition, the VA had a significant direct correlation with the MMSE showing that the VA diminished when cognitive ability decreased in AD. The predictive value of the VA was significant with a value of 71.4%. Although some authors did not find significant differences in VA in patients with AD [12,15,18–24], other authors found a VA loss [25,26], associated in some cases with visual hallucinations (13%), when the VA was gravely impaired [27,28].

The degeneration of the axons of the RGCs in the AD can selectively affect the magnocellular RGCs [29], which probably constitute the predominant projection of the superior colliculus (extraocular movements control) and the magnocellular layers of the lateral geniculate nucleus. In addition, the axons of the RGCs corresponding to the parvocellular pathway (detail) also diminished in the optic nerve in the AD [30]. In AD patients, more abundant Aβ plaques in the parvocellular layers were also identified compared to the magnocellular layers. There was also a presence of neurofibrillary tangles [31,32]. The lack of control of the extraocular movements link to the damaged parvocellular pathway could result in the VA decrease in our AD patients. In our study, patients with moderate AD exhibited a decrease in VA with respect to the control as they did in patients with mild AD. These moderate AD patients did not show significant differences in comparison with mild AD in spite of a loss of neurons in the retina. These findings could be due to adaptation phenomena and neural compensation [33].

CS in patients with mild AD and moderate AD was significantly reduced at all spatial frequencies. The most pronounced decrease occurred at the highest spatial frequencies. In comparison with the controls in the frequency of 18 cpd, the reduction was -40.67 in the mild AD group and -36.98% in the moderate AD group. This observation suggests that both groups (mild and moderate) suffered some damage in their function, both in the cells of the parvocellular and the magnocellular pathway. Apparently, the former cells were the most affected. The CS in moderate AD patients did not present significant changes with respect to mild AD. These findings suggest that the CS undergoes a steep decline in all spatial frequencies when the disease begins, and this loss stabilizes with the progression of the disease. This discovery is similar to what occurred in VA due to a possible adaptive phenomenon and neural compensation to maximize the transmission of information [33].

Most studies, including our work, found that the CS function was reduced in all spatial frequencies [12,15,16,20,25,34–37]. Some studies confirmed that the greatest reduction occurs in high spatial frequencies [16,34,38]. However, other studies showed that the greatest reduction occurs in the low spatial frequencies [14,15,21,24,39], and some did not find differences between the controls [18,23]. These discrepancies were possibly due to the different stages of AD patients and the tests used for the CS examination [13] since some tests are influenced by the VA value (Regan, Vistech VCTS-6500) while others are not influenced by it (Pelli Robson, Freiburg and CSV-100E). According to our results, a correlation between CS and retinal structural changes has been described [24], especially in the macular area. Our aROC values coincided with the most sensitive spatial frequencies, which were 18 cpg (83.3%), followed by 12 cpg (82.8%). These results showed that the CS, specifically when higher frequencies are used, has the best predictive value of all the psychophysical tests analysed. Therefore, CS is a sensitive tool to identify subclinical worsening of the visual function [40,41]. In addition, the correlation analysis in our work showed a strong statistical significance between all the spatial frequencies, and the MMSE test is a predictive test for the cognitive decline in the AD patients.

The colour perception in our patients did not show dyschromatopsia. However, when we analysed the total number of non-specific errors in performing the test, we found a significant increase of errors in moderate AD patients compared to those with mild AD (77,34%) and the controls (132,26%) both in the tritan region (blue) and in the deutan region (red-green). Some studies found no differences in colour perception between the AD patients and the control group [35,42,43]. However, in a more recent work using our colour test, (Farnsworth 28-hue) our result showed significant differences between patients with moderate AD and the controls [24]. Furthermore, in our study, patients with mild AD also presented significant differences in colour perception in the tritan and deutan axes with respect to the control. This fact and the results obtained in the aROC curves, with predictive values between 70–75%, lead us to postulate that chromatic vision examination could constitute a biomarker of the early diagnosis of AD. The analysis of correlations of non-specific errors of colour perception in the MMSE score showed that both the total errors and the nonspecific errors of the tritan and deutan region correlated significantly and inversely with the MMSE score of our AD patients. Results have been corroborated by other authors using different tests [15,25,44–48]. Nevertheless, the use of different tests in the analysis of chromatic vision could produce erroneous interpretations. With respect to the Ishihara test, in its realization, it is necessary to identify a pattern made of smaller pieces that differ in colour. One of the symptoms suffered by AD patients is the inability to see the whole of the parts (simultagnosia) due to a bilateral occipital-parietal dysfunction. Therefore, the Ishihara test would not be adequate to assess patients with AD. However, the realization of other tests like Farnsworth, based on the discrimination of tones, would not be affected by this problem [49].

Some authors have postulated that the problem of colour perception in patients with AD is not purely cognitive. Nevertheless, it seems to be related to damage in the structures responsible for the perception of colour stimuli [50]. The increase in nonspecific errors in the tritan and deutan axes observed in our AD patients suggests that, in addition to the loss of magno- and parvocellular-cells mentioned above, the koniocellular pathway could also be involved due to its association with the blue-yellow spectrum [51]. In addition, it has also been described that patients with AD have a significant reduction in the V4 area, which is a key region that processes colour vision [52,53]. On the other hand, degeneration in the photoreceptor layer has also been described in patients with AD, which is not restricted to a single cone type due to the decrease in melatonin and its antioxidant effects that occur in this pathology [54,55]. Melatonin and its antioxidant effects could be added to the photoreceptor degeneration induced by Aβ deposits in this layer, as seen in AD animal models [56,57]. In these diseases, a decrease in the area from the ellipsoids to the EPR was described by OCT, which showed a direct correlation with the degree of cognitive impairment [58].

With respect to PDT, some authors have observed a significant correlation between mild AD and MMSE values [10]. Our patients with mild AD and moderate AD failed to read a significantly larger number of PDT sheets compared to the control patients. This failure could be due to a disruption of the visual information processing at the brain level [59]. This processing would take place in the parietal and frontal regions, being mediated by the magnocellular pathway, which transmits information carrying the low frequencies of CS [60]. As we mentioned above, it has been described that magnocellular axons, which project to lateral geniculate nucleus, were affected starting from the early AD stages [29]. In our work, the PDT showed a high direct correlation with the MMSE scores. Furthermore, the values of the aROC curves showed a good prognostic value (71.4%). Thus, the PDT could be considered a good test for the examination of the visual superior processing in AD patients.

In our study involving moderate AD patients, when the peripapillary segmentation was performed in 4 sectors in the OCT, a significant decrease (-15.0%) in the lower sector was observed with respect to controls. The analysis of the correlations with the MMSE showed that both the upper and lower sectors had a significant direct correlation with the cognitive state of the patient. In comparison with the control subjects, it was reported that the eyes of the AD group showed a reduction of thickness in all peripapillary RNFL quadrants measured by OCT [61–68]. However, some OCT studies on peripapillary thickness found that the RNFL thinning was restricted to the superior quadrant [26,69–73] or in the superior and inferior quadrants [74–77]. These discrepancies could be due to different factors such as i) the inclusion of patients with different stages of cognitive deterioration in AD ii) the use of different OCT devices, with different segmentation of the software; and iii) sample selections of patients with little rigor, including patients with retinal abnormalities such as epiretinal membranes or drusen, which can influence the thickness of the retina. When we examined the peripapillary area by segmentation into 12 hourly sectors, the eyes of the moderate AD group showed a significant thinning in the superonasal and inferotemporal sectors of the papilla with respect to the controls, while in the patients with mild AD, there was a statistically significant decrease only in the hour sector 2. These sectors of the papilla are those that showed a better correlation with the MMSE score and the thinning the superonasal and inferotemporal sectors when the disease progressed. The aROC curves revealed that the hourly sector 2 and the hourly sector 8 had a high predictive value (71.5% and 66.8% respectively). Only in two studies were the peripapillary hourly sectors analysed in detail [62,78]. In one of the works, the patient's sample had a greater cognitive deterioration than ours; therefore, it is not comparable [62]. However, coinciding with our results, it has been observed that some peripapillary regions were thickened, and others were thinned, being indicative of dynamic changes in the RNFL of the peripapillary area [78].

Some studies showed a thinning of the retina associated with the advance of cognitive decline when it was correlated with the MMSE score [72,77,79–81], and it has also been observed that the peripapillary GCL-IPL complex has a strong correlation with the MMSE [67]. Moreover, in patients with mild AD (MMSE 22.7 ± 2.2), after 12 months of disease evolution, the peripapillary retinal thickness was significantly decreased in these patients with respect to the controls. This change was more prominent in the upper and lower regions, and the decrease in thickness was parallel to the cognitive decline [80]. It has been suggested that the lower quadrant peripapillary could be the area with greater specificity and sensitivity to detect cognitive decline in early stages of AD [72,74,80]. In our analysis, only this quadrant showed a significant correlation with the MMSE score (r = 0.328, p <0.01). This variability of results may be due to the different stages of the AD patients included in the studies, and to the methods used to analyse the peripapillary region because many authors segment the papilla only in 4 sectors, excluding the information provided by the 12 segmentation sectors.

The macular area, which was recently analysed, demonstrated a significant decrease in the thickness of this retinal region in patients with AD with respect to controls [24,62,71,76,77,82–87]. However, in some studies, when analysing the foveal area, a non-significant thickening was found in patients with AD [24,84]. In our study of macular thickness, both the analysis of the concentric circular sectors and macular analysis through a 6x6 square grid were carried out in detail because we consider that the information provided by the analysis of this region represents a great value, being easy to assess. We observed a foveal thickening (7.1%) in moderate AD, compared with mild AD, which indicates that there is a progression of the disease. In the analysis of the 6x6 square grid, the thickening, specifically of squares 5, 6, 11 and 21 in moderate AD with respect to mild AD, also indicated disease progression since we found significant thinning in the squares 6, 12,15, 21 and 22 in patients with mild AD. Aβ and pTau deposits, could produce the aforementioned thickening [88], which would induce an inflammation with the activation of the microglia [57], generating, in turn, a cytotoxic effect in the retinal neurons [57,89].

In studies using the latest OCT technology, which allows the separate analysis of the different retinal layers, we observed a thinning of the RNFL+GCL complex in AD patients, while the outer retinal layers were respected [77,79,83]. In addition, a significant reduction in the complex formed by the GCL and the IPL was described. A positive correlation between the thickness of the complex with the values of the MMSE in AD was established [90]. It was also postulated that this complex could have a higher prognostic value than the values of the peripapillary RNFL thickness, and it was related to the decrease in macular blood flow velocity [82]. This degeneration in the innermost layers of the retina was also observed in our mild AD with a marked thinning in the RNFL, GCL and IPL. The thinning of these internal retinal layers was also described after analysing 2124 patients from the Rotterdam study, linked to a smaller volume in the cerebral white and grey matter [91]. In addition, they observed that there was a significant relationship between the decrease in RNFL and the GCL with a smaller hippocampal volume, which is one of the typical clinical characteristics of AD [91].

In our mild AD patients, in addition to the thinning of some retinal layers, we observed an increase in thickness in INL and ONL, which could be associated with inflammatory processes and cellular movements in these outer layers. Moreover, some authors observed macular areas with significant thickening in the RNFL and in the GCL-IPL complex. These authors found that the areas of thinning in these two layers were adjacent to areas of thickening, suggesting that these layers can undergo dynamic changes during the progression of AD [78]. Other studies have reported a selective increase in the thickness of IPL related to Aβ deposits in the brain observed by PET in a preclinical stage of AD patients. In the longitudinal study of these patients at 27 months, they found a decrease in the volume of the IPL, INL and ONL, in addition to a thinning in the lower quadrant [92]. The authors explain that the reason for the increase in IPL in these patients is because acetylcholine is released by different types of amacrine, and possibly, by bipolar cells, being the cholinergic synapses concentrated in this layer [92–94]. Similarly, one of the earliest changes in the brain of AD patients is the neurochemical changes in the cholinergic system [95], and therefore, the synaptic pathology would be the first neurotoxic consequence of the progressive neurodegeneration in these patients [96].

On the whole, the findings of all these studies suggest that certain abnormalities in the brain could be reflected in the retina as the thinning of RNFL, GCL and IPL [97,98]. On the other hand, the ganglion cell apoptosis can cause anterograde degeneration leading to a thinner RNFL and occasionally cause smaller volumes of white and grey matter in the brain regions corresponding to the visual tract [99,100].

In conclusion, the first changes in mild AD patients appear in the psychophysical tests and in the central macula with a decrease of the central retinal thickness, which is the consequence of the degeneration of the inner layers of the retina. When there was a disease progression to moderate AD, psychophysical tests remained stable with respect to the decrease in mild AD. However, a significant thinning in the peripapillary retina and a thickening in the central retina appeared. Moreover, the thickening in the outer layers and in the INL was significant. The changes that appear in moderate AD compared with mild AD defined the progress of the disease correlating significantly with the cognitive decline. The presence of AD is best predicted based on CS at any frequency (though higher frequencies are better).

## Supporting information

S1 File Study’s underlying data set. (ZIP)

## References

[1] Sharma S, Lipincott W. Biomarkers in Alzheimer’s Disease-Recent Update. *Curr Alzheimer Res*. 2017;14: 1–1. 10.2174/1567205014666170220141822 28219319

[2] Ghiso JA, Doudevski I, Ritch R, Rostagno AA. Alzheimer’s disease and glaucoma: mechanistic similarities and differences. *J Glaucoma*. Department of Pathology, New York University Langone Medical Center, New York, NY, USA.; 2013;22 Suppl 5: S36–8. 10.1097/IJG.0b013e3182934af6 [doi] 23733125 PMC3955061

[3] Calabrese V, Scapagnini G, Giuffrida Stella AM, Bates TE, Clark JB. Mitochondrial involvement in brain function and dysfunction: relevance to aging, neurodegenerative disorders and longevity. *Neurochem Res*. Department of Chemistry, Faculty of Medicine, University of Catania, Italy. calabres@mbox.unict.it; 2001;26: 739–764. 11519733 10.1023/a:1010955807739

[4] Martínez-Lazcano JC, Boll-Woehrlen MC, Hernández-Melesio MA, Rubio-Osornio M, Sánchez-Mendoza MA, Ríos C. Radicales libres y estrés oxidativo en las enfermedades neurodegenerativas. *Mensaje Bioquim*. 2010;34: 43–59.

[5] de Lau LM, Breteler MM. Epidemiology of Parkinson’s disease. *Lancet Neurol*. 2006;5: 525–535. 10.1016/S1474-4422(06)70471-9 16713924

[6] Alberca Serrano R. Enfermedad de Alzheimer y otras demencias. 4a. Madrid: Médica Panamericana; 2010.

[7] Patton N, Aslam T, MacGillivray T, Pattie A, Deary IJ, Dhillon B. Retinal vascular image analysis as a potential screening tool for cerebrovascular disease: a rationale based on homology between cerebral and retinal microvasculatures. *J Anat*. Wiley Online Library; 2005;206: 319–348. 10.1111/j.1469-7580.2005.00395.x 15817102 PMC1571489

[8] Salobrar-Garcia E, De Hoz R, Rojas B, Ramirez AIAI, Salazar JJJJ, Yubero R, et al Ophthalmologic Psychophysical Tests Support OCT Findings in Mild Alzheimer’s Disease. *J Ophthalmol*. Hindawi Publishing Corporation; 2015;2015: Article ID 736949, 10 pages. 10.1155/2015/736949 PMC4461784 26106485

[9] Folstein MF, Folstein SE, McHugh PR. “Mini-mental state”: a practical method for grading the cognitive state of patients for the clinician. *J Psychiatr Res*. Pergamon; 1975;12: 189–198. 1202204 10.1016/0022-3956(75)90026-6

[10] Rami L, Serradell M, Bosch B, Villar A, Molinuevo JL. Perception Digital Test (PDT) for the assessment of incipient visual disorder in initial Alzheimer’s disease. *Neurologia*. Unidad Memoria-Alzheimer, Servicio de Neurologia, Institut Clinic de Neurociencies IDIBAPS, Hospital Clinic i Universitari, Barcelona.; 2007;22: 342–347. 17610161

[11] Roth A. Test-28 hue de Roth selon Farnsworth–Munsell (Manual). Luneau, Paris 1966; 5945791

[12] Lakshminarayanan V, Lagrave J, Kean ML, Dick M, Shankle R. Vision in dementia: contrast effects. *Neurol Res*. School of Optometry, University of Missouri-St. Louis, USA.; 1996;18: 9–15. 8714529 10.1080/01616412.1996.11740369

[13] Neargarder SA, Stone ER, Cronin-Golomb A, Oross S. The impact of acuity on performance of four clinical measures of contrast sensitivity in Alzheimer’s disease. *Journals Gerontol Ser B Psychol Sci Soc Sci*. Oxford University Press; 2003;58: P54–P62. 10.1093/geronb/58.1.p54 12496302

[14] Cronin-Golomb A, Gilmore GC, Neargarder S, Morrison SR, Laudate TM. Enhanced stimulus strength improves visual cognition in aging and Alzheimer’s disease. *Cortex*. Elsevier; 2007;43: 952–966. 10.1016/S0010-9452(08)70693-2 17941352

[15] Cronin-Golomb A, Corkin S, Rizzo JF, Cohen J, Growdon JH, Banks KS. Visual dysfunction in Alzheimer’s disease: relation to normal aging. *Ann Neurol*. Department of Brain and Cognitive Sciences, Massachusetts Institute of Technology, Cambridge.; 1991;29: 41–52. 10.1002/ana.410290110 1996878

[16] Gilmore GC, Whitehouse PJ. Contrast sensitivity in Alzheimer’s disease: A 1-year longitudinal analysis. *Optom Vis Sci*. 1995;72: 83–91. 7753532 10.1097/00006324-199502000-00007

[17] Berry SM. A Comparison Between Number and Letter Acuities Among Patients with Dementia. The University of Alabama 2017.

[18] Schlotterer G, Moscovitch M, Crapper-McLachlan D. Visual processing deficits as assessed by spatial frequency contrast sensitivity and backward masking in normal ageing and Alzheimer’s disease. *Brain*. ENGLAND; 1984;107: 309–325. 10.1093/brain/107.1.309 6697160

[19] Wright CE, Drasdo N, Harding GFAA. Pathology of the optic nerve and visual association areas information given by the flash and pattern visual evoked potential, and the temporal and spatial contrast sensitivity function. *Brain*. Oxford Univ Press; 1987;110: 107–120. 10.1093/brain/110.1.107 3801846

[20] Mendez MF, Tomsak RL, Remler B. Disorders of the visual system in Alzheimer’s disease. *J Neuro-Ophthalmology*. LWW; 1990;10: 62–69. 2139054

[21] Levine DN, Lee JM, Fisher CM. The visual variant of Alzheimer’s disease A clinicopathologic case study. *Neurology*. AAN Enterprises; 1993;43: 305 10.1212/wnl.43.2.305 8437694

[22] Martinelli V, Locatelli T, Comi G, Lia C, Alberoni M, Bressi S, et al Pattern Visual Evoked Potential Mapping in Alzheimers Disease Correlations with Visuospatial Impairment. *Dement Geriatr Cogn Disord*. Karger Publishers; 1996;7: 63–68. Available: http://www.ncbi.nlm.nih.gov/pubmed/8866677 10.1159/000106855 8866677

[23] Rizzo M, Nawrot M. Perception of movement and shape in Alzheimer’s disease. *Brain*. Oxford Univ Press; 1998;121: 2259–2270. 9874479 10.1093/brain/121.12.2259

[24] Polo V, Rodrigo MJ, Garcia-Martin E, Otin S, Larrosa JM, Fuertes MI, et al Visual dysfunction and its correlation with retinal changes in patients with Alzheimer’s disease. *Eye (Lond)*. Ophthalmology Department, Miguel Servet University Hospital, Zaragoza, Spain.; Aragon Health Research Institute (IIS Aragon-IACS), Zaragoza, Spain.; Ophthalmology Department, Miguel Servet University Hospital, Zaragoza, Spain.; Ophthalmology Dep(TRUNCATED; 2017; 10.1038/eye.2017.23 [doi]

[25] Sadun AA, Borchert M, DeVita E, Hinton DR, Bassi CJ. Assessment of visual impairment in patients with Alzheimer’s disease. *Am J Ophthalmol*. Elsevier; 1987;104: 113–120. 10.1016/0002-9394(87)90001-8 3618708

[26] Liu D, Zhang L, Li Z, Zhang XX, Wu Y, Yang H, et al Thinner changes of the retinal never fiber layer in patients with mild cognitive impairment and Alzheimer’s disease. *BMC Neurol*. BioMed Central Ltd; 2015;15: 14 10.1186/s12883-015-0268-6 25886372 PMC4342899

[27] Leroi I, Voulgari A, Breitner JCS, Lyketsos CG. The Epidemiology of Psychosis in Dementia. *Am J Geriatr Psychiatry*. Elsevier; 2003;11: 83–91. 10.1097/00019442-200301000-00011 12527543

[28] Bernardin F, Schwan R, Lalanne L, Ligier F, Angioi-Duprez K, Schwitzer T, et al The role of the retina in visual hallucinations: A review of the literature and implications for psychosis. *Neuropsychologia*. 2017;99: 128–138. 10.1016/j.neuropsychologia.2017.03.002 28263800

[29] Sadun AA, Bassi CJ. Optic nerve damage in Alzheimer’s disease. *Ophthalmology*. 1990;97: 9–17. 10.1016/s0161-6420(90)32621-0 2314849

[30] Syed AB, Armstrong RA, Smith CU. A quantitative analysis of optic nerve axons in elderly control subjects and patients with Alzheimer’s disease. *Folia Neuropathol*. 2005;43: 1–6. 15827884

[31] Leuba G, Saini K. Pathology of subcortical visual centres in relation to cortical degeneration in Alzheimer’s disease. *Neuropathol Appl Neurobiol*. Wiley Online Library; 1995;21: 410–422. 8632836 10.1111/j.1365-2990.1995.tb01078.x

[32] Leuba G, Saini K, Zimmermann V, Giannakopoulos P, Bouras C. Mild amyloid pathology in the primary visual system of nonagenarians and centenarians. *Dement Geriatr Cogn Disord*. Karger Publishers; 2001;12: 146–152. 10.1159/000051249 11173888

[33] Webster MA. Evolving concepts of sensory adaptation. *F1000 Biol Rep*. Faculty of 1000 Ltd; 2012;4: 21 10.3410/B4-21 23189092 PMC3501690

[34] Trick GL, Barris MC, Bickler‐Bluth M. Abnormal pattern electroretinograms in patients with senile dementia of the Alzheimer type. *Ann Neurol*. Wiley Online Library; 1989;26: 226–231. 10.1002/ana.410260208 2774510

[35] Bassi CJ, Solomon K, Young D. Vision in aging and dementia. *Optom Vis Sci*. LWW; 1993;70: 809–813. 8247482 10.1097/00006324-199310000-00005

[36] Cormack FK, Tovee M, Ballard C. Contrast sensitivity and visual acuity in patients with Alzheimer’s disease. *Int J Geriatr Psychiatry*. Wiley Online Library; 2000;15: 614–620. 10918342 10.1002/1099-1166(200007)15:7<614::aid-gps153>3.0.co;2-0

[37] Crow RW, Levin LB, LaBree L, Rubin R, Feldon SE. Sweep visual evoked potential evaluation of contrast sensitivity in Alzheimer’s dementia. *Invest Ophthalmol Vis Sci*. ARVO; 2003;44: 875–878. 10.1167/iovs.01-1101 12556424

[38] Hutton JT, Morris JL, Elias JW, Poston JN. Contrast sensitivity dysfunction in Alzheimer’s disease. *Neurology*. AAN Enterprises; 1993;43: 2328 10.1212/wnl.43.11.2328 8232951

[39] Baker DR, Mendez MF, Townsend JC, Ilsen PF, Bright DC. Optometric management of patients with Alzheimer’s disease. *J Am Optom Assoc*. 1997;68: 483–494. 9279048

[40] Marmor MF. Contrast sensitivity versus visual acuity in retinal disease. *Br J Ophthalmol*. 1986;70: 553–9. Available: 10.1136/bjo.70.7.553 3487345 PMC1041066

[41] Ward ME, Gelfand JM, Lui L-Y, Ou Y, Green AJ, Stone K, et al Reduced contrast sensitivity among older women is associated with increased risk of cognitive impairment. *Ann Neurol*. 2018;83: 730–738. 10.1002/ana.25196 29518257 PMC5947874

[42] Wood S, Mortel KF, Hiscock M, Breitmeyer BG, Caroselli JS. Adaptive and maladaptive utilization of color cues by patients with mild to moderate Alzheimer’s Disease. *Arch Clin Neuropsychol*. Elsevier; 1997;12: 483–489. 14590678

[43] Massoud F, Chertkow H, Whitehead V, Overbury O, Bergman H. Word-reading thresholds in Alzheimer disease and mild memory loss: a pilot study. *Alzheimer Dis Assoc Disord*. LWW; 2002;16: 31–39. Available: http://www.ncbi.nlm.nih.gov/pubmed/11882747 11882747 10.1097/00002093-200201000-00005

[44] Cronin-Golomb A, Sugiura R, Corkin S, Growdon JH. Incomplete achromatopsia in Alzheimer’s disease. *Neurobiol Aging*. Elsevier; 1993;14: 471–477. 8247229 10.1016/0197-4580(93)90105-k

[45] Kurylo DD, Corkin S, Dolan RP, Rizzo JF, Parker SW, Growdon JH. Broad-band visual capacities are not selectively impaired in Alzheimer’s disease. *Neurobiol Aging*. Elsevier; 1994;15: 305–311. Available: http://www.ncbi.nlm.nih.gov/pubmed/7936054 7936054 10.1016/0197-4580(94)90025-6

[46] Cronin-Golomb A, Corkin S, Growdon JH. Visual dysfunction predicts cognitive deficits in Alzheimer’s disease. *Optom Vis Sci*. LWW; 1995;72: 168–176. 7609939 10.1097/00006324-199503000-00004

[47] Wijk H, Berg S, Sivik L, Steen B. Colour discrimination, colour naming and colour preferences among individuals with Alzheimer’s disease. *Int J Geriatr Psychiatry*. Wiley Online Library; 1999;14: 1000–1005. Available: http://www.ncbi.nlm.nih.gov/pubmed/10607966 10607966

[48] Rizzo JF III, Cronin-Golomb A, Growdon JH, Corkin S, Rosen TJ, Sandberg MA, et al Retinocalcarine function in Alzheimer’s disease: a clinical and electrophysiological study. *Arch Neurol*. Am Med Assoc; 1992;49: 93–101. 10.1001/archneur.1992.00530250097023 1728270

[49] Pelak VS. Ocular motility of aging and dementia. *Curr Neurol Neurosci Rep*. Department of Neurology, University of Colorado School of Medicine, Aurora, CO 80045, USA. victoria.pelak@ucdenver.edu; 2010;10: 440–447. 10.1007/s11910-010-0137-z 20697981

[50] Salamone G, Di Lorenzo C, Mosti S, Lupo F, Cravello L, Palmer K, et al Color discrimination performance in patients with Alzheimer’s disease. *Dement Geriatr Cogn Disord*. Karger Publishers; 2009;27: 501–507. 10.1159/000218366 19451717

[51] Martin PR, White AJR, Goodchild AK, Wilder HD, Sefton AE. Evidence that Blue‐on Cells are Part of the Third Geniculocortical Pathway in Primates. *Eur J Neurosci*. Wiley Online Library; 1997;9: 1536–1541. 9240412 10.1111/j.1460-9568.1997.tb01509.x

[52] Chan D, Crutch SJ, Warrington EK. A disorder of colour perception associated with abnormal colour after-images: a defect of the primary visual cortex. *J Neurol Neurosurg Psychiatry*. 2001;71: 515–517. 10.1136/jnnp.71.4.515 11561036 PMC1763505

[53] Brewer AA, Barton B. Changes in Visual Cortex in Healthy Aging and Dementia.

[54] Savaskan E, Wirz-Justice A, Olivieri G, Pache M, Krauchi K, Brydon L, et al Distribution of melatonin MT1 receptor immunoreactivity in human retina. *J Histochem Cytochem*. Department of Gerontopsychiatry, Psychiatric University Clinic, University of Basel, Wilhelm Klein-Strasse 27, CH-4025 Basel, Switzerland. esavaskan@datacomm.ch; 2002;50: 519–526. 10.1177/002215540205000408 11897804

[55] Marchiafava PL, Longoni B. Melatonin as an antioxidant in retinal photoreceptors. *J Pineal Res*. marchiafava@dfb.unipi.it; 1999;26: 184–189. 10231733 10.1111/j.1600-079x.1999.tb00582.x

[56] Hart NJ, Koronyo Y, Black KL, Koronyo-Hamaoui M. Ocular indicators of Alzheimer’s: exploring disease in the retina. *Acta Neuropathol*. Springer Berlin Heidelberg; 2016;132: 767–787. 10.1007/s00401-016-1613-6 27645291 PMC5106496

[57] Bruban J, Glotin AL, Dinet V, Chalour N, Sennlaub F, Jonet L, et al Amyloid-beta(1–42) alters structure and function of retinal pigmented epithelial cells. *Aging Cell*. Centre de Recherche des Cordeliers, Universite Pierre et Marie Curie—Paris 6, UMR S 872, F-75006 Paris, France.; 2009;8: 162–177. 10.1111/j.1474-9726.2009.00456.x 19239420

[58] Uchida A, Pillai JA, Bermel R, Bonner-Jackson A, Rae-Grant A, Fernandez H, et al Outer Retinal Assessment Using Spectral-Domain Optical Coherence Tomography in Patients With Alzheimer’s and Parkinson’s Disease. *Invest Ophthalmol Vis Sci*. 2018;59: 2768–2777. 10.1167/iovs.17-23240 29860463 PMC5983910

[59] Saumier D, Chertkow H, Arguin M, Whatmough C. Establishing visual category boundaries between objects: a PET study. *Brain Cogn*. 2005;59: 299–302. 10.1016/j.bandc.2004.02.060 15882921

[60] Bar M. A cortical mechanism for triggering top-down facilitation in visual object recognition. *J Cogn Neurosci*. 2003;15: 600–9. 10.1162/089892903321662976 12803970

[61] Paquet C, Boissonnot M, Roger F, Dighiero P, Gil R, Hugon J. Abnormal retinal thickness in patients with mild cognitive impairment and Alzheimer’s disease. *Neurosci Lett*. Elsevier; 2007;420: 97–99. 10.1016/j.neulet.2007.02.090 17543991

[62] Iseri PK, Altinas Ö, Tokay T, Yüksel N. Relationship between cognitive impairment and retinal morphological and visual functional abnormalities in Alzheimer disease. *J neuro-ophthalmology*. 2006;26: 18. 10.1097/01.wno.0000204645.56873.26 16518161

[63] Valenti DA. Neuroimaging of retinal nerve fiber layer in AD using optical coherence tomography. *Neurology*. AAN Enterprises; 2007;69: 1060 10.1212/01.wnl.0000280584.64363.83 17785676

[64] Moreno-Ramos T, Benito-León J, Villarejo A, Bermejo-Pareja F. Retinal Nerve Fiber Layer Thinning in Dementia Associated with Parkinson’s Disease, Dementia with Lewy Bodies, and Alzheimer’s Disease. *J Alzheimer’s Dis*. IOS Press; 2013;34: 659–664. 23271313 10.3233/JAD-121975

[65] He XF, Liu YT, Peng C, Zhang F, Zhuang S, Zhang JS. Optical coherence tomography assessed retinal nerve fiber layer thickness in patients with Alzheimer’s disease: a meta-analysis. *Int J Ophthalmol*. Press of International Journal of Ophthalmology; 2012;5: 401–405. 10.3980/j.issn.2222-3959.2012.03.30 22773997 PMC3388417

[66] Parisi V, Restuccia R, Fattapposta F, Mina C, Bucci MG, Pierelli F. Morphological and functional retinal impairment in Alzheimer’s disease patients. *Clin Neurophysiol*. Elsevier; 2001;112: 1860–1867. 11595144 10.1016/s1388-2457(01)00620-4

[67] Ferrari L, Huang SC, Magnani G, Ambrosi A, Comi G, Leocani L. Optical Coherence Tomography Reveals Retinal Neuroaxonal Thinning in Frontotemporal Dementia as in Alzheimer’s Disease. *J Alzheimers Dis*. Department of Neurology and INSPE—Institute of Experimental Neurology, San Raffaele Scientific Institute, Milan, Italy.; University Vita-Salute San Raffaele Milan, Italy.; Department of Neurology and INSPE—Institute of Experimental Neurology(TRUNCATED; 2017;56: 1101–1107. 10.3233/JAD-160886 28106555

[68] Ko F, Muthy ZA, Gallacher J, Sudlow C, Rees G, Yang Q, et al Association of Retinal Nerve Fiber Layer Thinning With Current and Future Cognitive Decline: A Study Using Optical Coherence Tomography. *JAMA Neurol*. 2018;75: 1198–1205. 10.1001/jamaneurol.2018.1578 29946685 PMC6233846

[69] Berisha F, Feke GT, Trempe CL, McMeel JW, Schepens CL. Retinal abnormalities in early Alzheimer’s disease. *Invest Ophthalmol Vis Sci*. ARVO; 2007;48: 2285–2289. 10.1167/iovs.06-1029 17460292

[70] Kromer R, Serbecic N, Hausner L, Froelich L, Aboul-enein F. Detection of retinal nerve fiber layer defects in Alzheimer ‘ s disease using SD-OCT. *Front Psychiatry*. 2014;5: 1–7. 10.3389/fpsyt.2014.00001 24616709 PMC3934110

[71] Chi Y, Wang YH, Yang L. The investigation of retinal nerve fiber loss in Alzheimer’s disease. *Zhonghua Yan Ke Za Zhi*. Department of Ophthalmology, Peking University First Hospital, Beijing 100034, China.; 2010;46: 134–139. 10.3760/cma.j.issn.04124081.2010.02.009 20388347

[72] Shen Y, Shi Z, Jia R, Zhu Y, Cheng Y, Feng W, et al The attenuation of retinal nerve fiber layer thickness and cognitive deterioration. *Front Cell Neurosci*. Frontiers Media SA; 2013;7: 1–7. 10.3389/fncel.2013.00001 24065883 PMC3777215

[73] Kirbas S, Turkyilmaz K, Anlar O, Tufekci A, Durmus M. Retinal nerve fiber layer thickness in patients with Alzheimer disease. *J Neuro-Ophthalmology*. LWW; 2013;33: 58–61. 10.1097/WNO.0b013e318267fd5f 22918296

[74] Kesler A, Vakhapova V, Korczyn AD, Naftaliev E, Neudorfer M. Retinal thickness in patients with mild cognitive impairment and Alzheimer’s disease. *Clin Neurol Neurosurg*. Elsevier; 2011;113: 523–526. 10.1016/j.clineuro.2011.02.014 21454010

[75] Lu Y, Li Z, Zhang X, Ming B, Jia J, Wang R, et al Retinal nerve fiber layer structure abnormalities in early Alzheimer’s disease: evidence in optical coherence tomography. *Neurosci Lett*. Department of Ophthalmology, Xuan Wu Hospital, Capital Medical University, Beijing 100053, China. louiseluyan@yahoo.com.cn: Elsevier Ireland Ltd; 2010;480: 69–72. 10.1016/j.neulet.2010.06.006 20609426

[76] M Moschos M, Markopoulos I, Chatziralli I, Rouvas A, G Papageorgiou S, Ladas I, et al Structural and Functional Impairment of the Retina and Optic Nerve in Alzheimer’s Disease. *Curr Alzheimer Res*. 2012;9: 782–788. 10.2174/156720512802455340 22698074

[77] Cunha LP, Lopes LC, Costa-Cunha LVFVF, Costa CF, Pires LAA, Almeida ALM, et al Macular Thickness Measurements with Frequency Domain-OCT for Quantification of Retinal Neural Loss and its Correlation with Cognitive Impairment in Alzheimer’s Disease. *PLoS One*. Public Library of Science; 2016;11: e0153830 10.1371/journal.pone.0153830 27104962 PMC4841530

[78] Lad EM, Mukherjee D, Stinnett SS, Cousins SW, Potter GG, Burke JR, et al Evaluation of inner retinal layers as biomarkers in mild cognitive impairment to moderate Alzheimer’s disease. Paul F, editor. *PLoS One*. 2018;13: e0192646 10.1371/journal.pone.0192646 29420642 PMC5805310

[79] Bayhan HA, Aslan Bayhan S, Celikbilek A, Tanık N, Gürdal C, Tanik N, et al Evaluation of the chorioretinal thickness changes in Alzheimer’s disease using spectral‐domain optical coherence tomography. *Clin Experiment Ophthalmol*. Wiley Online Library; 2014;43: 145–151. 10.1111/ceo.12386 24995484

[80] Trebbastoni A, D’Antonio F, Bruscolini A, Marcelli M, Cecere M, Campanelli A, et al Retinal nerve fibre layer thickness changes in Alzheimer’s disease: Results from a 12-month prospective case series. *Neurosci Lett*. Elsevier Ireland Ltd; 2016;629: 165–170. 10.1016/j.neulet.2016.07.006 27394689

[81] Oktem EO, Derle E, Kibaroglu S, Oktem C, Akkoyun I, Can U. The relationship between the degree of cognitive impairment and retinal nerve fiber layer thickness. *Neurol Sci*. Springer; 2015; 1–6. 10.1007/s10072-015-2188-z 25575807

[82] Choi SH, Park SJ, Kim NR. Macular Ganglion Cell -Inner Plexiform Layer Thickness Is Associated with Clinical Progression in Mild Cognitive Impairment and Alzheimers Disease. *PLoS One*. Department of Neurology, Inha University School of Medicine, Incheon, Korea.; Department of Ophthalmology and Inha Vision Science Laboratory, Inha University School of Medicine, Incheon, Korea.; Department of Ophthalmology and Inha Vision Scienc(TRUNCATED; 2016;11: e0162202 10.1371/journal.pone.0162202 27598262 PMC5012569

[83] Marziani E, Pomati S, Ramolfo P, Cigada M, Giani A, Mariani C, et al Evaluation of retinal nerve fiber layer and ganglion cell layer thickness in Alzheimer’s disease using spectral-domain optical coherence tomography. *Invest Ophthalmol Vis Sci*. Eye Clinic, Department of Biomedical and Clinical Sciences Luigi Sacco, University of Milan, Milan, Italy.; 2013;54: 5953–5958. 10.1167/iovs.13-12046 23920375

[84] Garcia-Martin ES, Rojas B, Ramirez AI, de Hoz R, Salazar JJ, Yubero R, et al Macular Thickness as a Potential Biomarker of Mild Alzheimer’s Disease. *Ophthalmology*. Instituto de Investigaciones Oftalmologicas Ramon Castroviejo, Universidad Complutense de Madrid, Madrid, Spain.; Instituto de Investigaciones Oftalmologicas Ramon Castroviejo, Universidad Complutense de Madrid, Madrid, Spain; Facultad de Medici(TRUNCATED; 2014;121: 1149–1151. 10.1016/j.ophtha.2013.12.023 24656417

[85] Ascaso FJ, Cruz N, Modrego PJ, Lopez-Anton R, Santabárbara J, Pascual LF, et al Retinal alterations in mild cognitive impairment and Alzheimer’s disease: an optical coherence tomography study. *J Neurol*. Springer; 2014; 1–9. 10.1007/s00415-014-7374-z 24846203

[86] Gao L, Liu Y, Li X, Bai Q, Liu P. Abnormal retinal nerve fiber layer thickness and macula lutea in patients with mild cognitive impairment and Alzheimer’s disease. *Arch Gerontol Geriatr*. Elsevier; 2015;60: 162–167. 10.1016/j.archger.2014.10.011 25459918

[87] Giménez-Castejón D, Gómez-Gallego M, Martínez-Martínez ML, Dudekova M, Lajara-Blesa J. ¿ Hasta dónde llega la precocidad de la tomografía de coherencia óptica en el deterioro cognitivo? *Rev Neurol*. 2016;63: 5–10. 27345274

[88] Grimaldi A, Brighi C, Peruzzi G, Ragozzino D, Bonanni V, Limatola C, et al Inflammation, neurodegeneration and protein aggregation in the retina as ocular biomarkers for Alzheimer’s disease in the 3xTg-AD mouse model. *Cell Death Dis*. 2018;9: 685 10.1038/s41419-018-0740-5 29880901 PMC5992214

[89] Lambert MP, Barlow AK, Chromy BA, Edwards C, Freed R, Liosatos M, et al Diffusible, nonfibrillar ligands derived from Abeta1-42 are potent central nervous system neurotoxins. *Proc Natl Acad Sci U S A*. 1998;95: 6448–53. Available: 10.1073/pnas.95.11.6448 9600986 PMC27787

[90] Jiang H, Wei Y, Shi Y, Wright CB, Sun X, Gregori G, et al Altered Macular Microvasculature in Mild Cognitive Impairment and Alzheimer Disease. *J Neuroophthalmol*. Department of Ophthalmology (HJ, YW, YS, GG, FZ, EAV, BLL, JW), Bascom Palmer Eye Institute, Miller School of Medicine, University of Miami, Miami, Florida; Department of Neurology (HJ, CBW, XS, TR), Evelyn F. McKnight Brain Institute, Universit(TRUNCATED; 2017; 10.1097/WNO.0000000000000580 [doi]

[91] Mutlu U, Bonnemaijer PWMM, Ikram MKAKA, Colijn JM, Cremers LGMM, Buitendijk GHSS, et al Retinal neurodegeneration and brain MRI markers: the Rotterdam Study. *Neurobiol Aging*. Department of Epidemiology, Erasmus University Medical Center, Rotterdam, the Netherlands; Department of Ophthalmology, Erasmus University Medical Center, Rotterdam, the Netherlands.; Department of Epidemiology, Erasmus University Medical Center(TRUNCATED: Elsevier Inc; 2017; https://doi.org/S0197-4580(17)30296-8 [pii]

[92] Santos CY, Johnson LN, Sinoff SE, Festa EK, Heindel WC, Snyder PJ. Change in retinal structural anatomy during the preclinical stage of Alzheimer’s disease. *Alzheimer’s Dement (Amsterdam*, *Netherlands)*. 2018;10: 196–209. 10.1016/j.dadm.2018.01.003 PMC5956814 29780864

[93] Masland RH, Mills JW. Autoradiographic identification of acetylcholine in the rabbit retina. *J Cell Biol*. 1979;83: 159–78. Available: 10.1083/jcb.83.1.159 92476 PMC2110447

[94] Massey SC, Neal MJ. The light evoked release of acetylcholine from the rabbit retina iN vivo and its inhibition by gamma-aminobutyric acid. *J Neurochem*. 1979;32: 1327–9. Available: 10.1111/j.1471-4159.1979.tb11062.x 430091

[95] Bowen DM, Benton JS, Spillane JA, Smith CC, Allen SJ. Choline acetyltransferase activity and histopathology of frontal neocortex from biopsies of demented patients. *J Neurol Sci*. 1982;57: 191–202. Available: 10.1016/0022-510x(82)90026-0 7161618

[96] Yoshiyama Y, Higuchi M, Zhang B, Huang S-M, Iwata N, Saido TC, et al Synapse loss and microglial activation precede tangles in a P301S tauopathy mouse model. *Neuron*. 2007;53: 337–51. 10.1016/j.neuron.2007.01.010 17270732

[97] Arnold SE, Hyman BT, Flory J, Damasio AR, Van Hoesen GW. The topographical and neuroanatomical distribution of neurofibrillary tangles and neuritic plaques in the cerebral cortex of patients with Alzheimer’s disease. *Cereb Cortex*. Oxford Univ Press; 1991;1: 103–116. 10.1093/cercor/1.1.103 1822725

[98] Tiraboschi P, Hansen LA, Thal LJ, Corey-Bloom J. The importance of neuritic plaques and tangles to the development and evolution of AD. *Neurology*. Dipartimento di Scienze Neurologiche, Ospedale Niguarda Ca’ Granda, Milan, Italy.; 2004;62: 1984–1989. 10.1212/01.wnl.0000129697.01779.0a 15184601

[99] Ohno-Matsui K. Parallel findings in age-related macular degeneration and Alzheimer’s disease. *Prog Retin Eye Res*. Department of Ophthalmology and Visual Science, Tokyo Medical and Dental University, Yushima, Bunkyo-ku, Japan. k.ohno.oph@tmd.ac.jp: Elsevier Ltd; 2011;30: 217–238. 10.1016/j.preteyeres.2011.02.004 21440663

[100] Kim M, Park KH, Kwon JW, Jeoung JW, Kim T-W, Kim DM. Retinal Nerve Fiber Layer Defect and Cerebral Small Vessel Disease. *Investig Opthalmology Vis Sci*. 2011;52: 6882 10.1167/iovs.11-7276 21791593

